# Reports on Cities, Towns, & Districts

**Published:** 1855-12

**Authors:** 


### SANITARY AND SOCIAL SCIENCE.

### REPORTS ON CITIES, TOWNS, & DISTRICTS.

### THE AVERAGE MORTALITY AND PUBLIC HEALTH OF THE HASTINGS DISTRICT.

While the public health of every town or village concerns its own inhabitants, there are certain places whose sanitary condition may be supposed, for various reasons, to be interesting to the whole community. Hastings is one of these, on account of the great number of invalids who annually resort thither, especially during the winter season; and therefore the public may like to know what is the average mortality of the place, and what the average state of the public health.

The following observations are founded almost entirely upon the Registers of Deaths in the Hastings District, which the Registrar General has, with his usual courtesy, allowed the writer to examine. The Hastings District* comprehends the four neighbouring villages, Pett, Guestling, Fairlight, and Ore; and it has been thought better to include these in the following calculations relative to the mortality of the place, partly in order to facilitate the comparison with other districts, and partly because the parish of Ore contains the Hastings Union Workhouse, which it seemed to be unfair to exclude. In 1851 the Hastings District contained 21,215 inhabitants, of whom 3594 lived in these four villages: the males were 9786; the females 11,429. The number of inhabitants at the present time (1855) is probably between 24,000 and 25,000.

Dr. Mackness, in his work on *Hastings considered as a Resort for Invalids,* does not give the average annual mortality. Mr. Creasy, in his *Report to the General Board of Health, etc.*, p. 31, reckons it at 18·7 per 1000, on an average of the seven years 1838-44, excluding the Ore Sub-District. In the *Registrar General's Ninth Annual Report* (Appendix, p. 199), the annual mortality in the Rye, Hastings, and Battle Districts, during the same seven years, is stated to have been 18·73 among the males, and 17·79 among the females. In the Appendix to the *Report on Public Health Bill, etc.*, p. 208, Dr. Farr states, that out of 222 Town Districts, there was one (Holyhead) in which the annual rate of mortality in the ten years 1841-50 was 16 in 1000, two (Lewisham and Keswick) in which it was 17 in·1000, and seven in which it was 18 in 1000: one of these seven towns was Hastings. Dr. Farr also states, that in the smaller country towns the rate of mortality is about 22 in 1000 (§ 1578).

With respect to the average rate of mortality in Hastings, the writer's own calculations agree very closely with the above state-

* For the information of strangers, it may be mentioned that "St. Leonards" (which sometimes signifies a parish, sometimes a township, according to Act of Parliament, and sometimes a post-town), forms part of the sub-district of St. Mary in-the-Castle.

ments. In the ten years 1845-54, the rate of mortality may be reckoned at either 18·39 in 1000 or 18·87 in 1000, according to the method of estimating the population; the latter estimate (in the writer's opinion) being probably the more correct, and therefore adopted here. The highest rate of mortality was 25·36 per 1000 in 1849 (the cholera year); the lowest was 15·80 per 1000 in 1854. In males the highest mortality was 28·23 per 1000 in 1849; the lowest was 16·57 per 1000 in 1854; the average was 20·32 per 1000. In females the highest mortality was 22·96 per 1000 in 1849; the lowest was 15·14 per 1000 in 1854; the average was 17·63 per 1000.

*Annual Rate of Mortality in the Hastings District.*

| Year. | MALES. | | | FEMALES. | | | BOTH SEXES. | | |
|---|---|---|---|---|---|---|---|---|---|
| | Estimated Population. | Deaths | Rate per 1000. | Estimated Population. | Deaths | Rate per 1000. | Estimated Population. | Deaths | Rate per 1000. |
| 1845 | 7379 | 140 | 18·97 | 8841 | 140 | 15·83 | 16,221 | 280 | 17·27 |
| 1846 | 7672 | 137 | 17·85 | 9168 | 146 | 15·92 | 16,841 | 283 | 16·81 |
| 1847 | 8018 | 168 | 20·95 | 9551 | 169 | 17·70 | 17,570 | 337 | 19·18 |
| 1848 | 8387 | 154 | 18·36 | 9931 | 186 | 18·74 | 18,319 | 340 | 18·56 |
| 1849 | 8821 | 249 | 28·23 | 10,373 | 238 | 22·96 | 19,195 | 487 | 25·36 |
| 1850 | 9225 | 228 | 24·72 | 10,835 | 212 | 19·55 | 20,061 | 440 | 21·94 |
| 1851 | 9786 | 218 | 22·28 | 11,429 | 198 | 17·33 | 21,215 | 416 | 19·61 |
| 1852 | 10,228 | 183 | 17·79 | 11,976 | 190 | 15·86 | 22,264 | 373 | 16·75 |
| 1853 | 10,792 | 189 | 17·52 | 12,481 | 216 | 17·31 | 23,273 | 405 | 17·41 |
| 1854 | 11,234 | 186 | 16·57 | 12,954 | 196 | 15·15 | 24,188 | 382 | 15·80 |
| Average . . . | | 185·2 | 20·32 | | 189·1 | 17·63 | | 374·3 | 18·87 |

There is less difference than might have been expected, in the rate of mortality in the three sub-districts. We should be prepared to find the fewest deaths in the rural sub-districts of Ore; but the mortality is in fact rather higher in this than in either of the other sub-districts, being 19·61 per 1000. This is caused by the deaths in the Union Workhouse, which is situated in this sub-district. Again, we should have expected to find the mortality in the sub-district of All Saints, which is the oldest and poorest part of the town, higher than in the newer and more fashionable sub-district of St. Mary-in-the-Castle; but the reverse of this is the case, the deaths in the former sub-district being 18·48 per 1000, and those in the latter sub-district being 19·00 per 1000. This is occasioned by the large number of visitors who die at Hastings, most of whom lodge in the better parts of the town.

The manner in which the deaths in a place are distributed throughout the year, is a sanitary test of some importance. In the Registrar-General's *Report on Cholera*, p. 49, it is stated, as a general result of many observations in Europe, " that, whenever the mortality of the six months after the summer solstice is much greater than the mortality of the first six months in the year, the absolute mortality of the place is high." The average quarterly mortality in the Hastings district is as follows:

| | | | |
|---|---|---|---|
| First Quarter . . | 101·1 | Third Quarter . . | 82·0 |
| Second Quarter . | 97·9 | Fourth Quarter . . | 93·6 |
| | 199·0 | | 175·6 |

*Average Mortality in each Month and each Season, with the mean height of Barometer, Temperature of the Air, amount of Rain, etc.*

| | Barometer. | Thermometer. | RAIN. | | DEATHS. | | |
|---|---|---|---|---|---|---|---|
| | | | Number of Days. | Amount in inches. | Number uncorrected. | Number corrected.* | Rate per 1000 of Population. |
| December . . | 29·62 | 41·60 | 14·22 | 2·38 | 35·0 | 34·38 | 20·80 |
| January . . | 29·62 | 37·63 | 14·44 | 2·91 | 33·1 | 32·50 | 19·66 |
| February . . | 29·69 | 38·16 | 11·66 | 2·20 | 28·3 | 30·50 | 18·45 |
| March . . . | 29·89 | 41·89 | 11·66 | 2·99 | 39·4 | 38·69 | 23·41 |
| April . . . | 2·975 | 47·40 | 7·33 | 1·20 | 36·4 | 36·94 | 22·36 |
| May . . . . | 29·80 | 53.76 | 11·02 | 1·87 | 32·2 | 31·62 | 19·13 |
| June . . . | 29·91 | 61·55 | 9·77 | 1·52 | 29·2 | 29·63 | 17·93 |
| July . . . . | 29·71 | 61·08 | 13·00 | 2·39 | 24·8 | 24·35 | 14·73 |
| August . . . | 29·84 | 59·22 | 12·10 | 2·36 | 25·5 | 25·03 | 15·15 |
| September . | 29·69 | 57·46 | 13·33 | 3·30 | 31·8 | 32·27 | 19·53 |
| October . . | 29·52 | 55·50 | 16·80 | 4·41 | 31·0 | 30·45 | 18·43 |
| November . . | 29·71 | 53·51 | 20·10 | 4·45 | 28·5 | 28·92 | 17·50 |
| Winter . . . | 29·64 | 39·13 | 40·32 | 7·49 | 95·4 | 96·51 | 19·45 |
| Spring . . . | 29·81 | 47·68 | 30·01 | 6·06 | 108·0 | 107·20 | 21·62 |
| Summer . . | 29·82 | 60·61 | 34·87 | 6·27 | 79·5 | 78·91 | 15·92 |
| Autumn . . | 29·64 | 55·15 | 50·23 | 12·16 | 90·4 | 90·72 | 18·29 |
| Whole Year . | 29·72 | 50·62 | 155·43 | 31·98 | 374·30 | 374·30 | 18·87 |
| Quarter of Year | — | — | 38·86 | 7·99 | 93·58 | 93·58 | — |
| Twelfth of Year | — | — | 12·95 | 2·66 | 31·19 | 31·19 | — |

The natural and philosophical division of the year into *seasons* is, for all medical and sanitary purposes, more important than the arbitrary and artificial division into *quarters*. It will be sufficiently accurate to consider the three coldest months (viz., December, January, and February) as *winter*, and the three hottest (viz., June, July, and August) as *summer*. The average mortality was least during the summer (viz., 15·92 per 1000), and gradually increased in the autumn (18·29 per 1000) and winter (19·45 per 1000), till it reached the maximum in spring (viz., 21·62 per 1000). It may be stated generally, that from December to May (with the exception

* In this column each month is considered to consist of 30·44 days, and each quarter of 91·31 days.

N.B. The Meteorological Tables are taken, with the correction of a few errors, from Mackness on *Hastings considered as a Resort for Invalids* (2nd ed.) They are not entirely satisfactory, but they are the best that at present exist.

of February) the rate of mortality is higher than the average; and that from June to November (with the exception of September) the mortality is below the average. Of all the months in the year, the highest rate of mortality prevailed in March (23·41 per 1000), and the lowest in July (14·73 per 1000).

In each season, the greatest number of deaths was caused by tubercular diseases (especially phthisis), and zymotic diseases. The mortality from the former class varied but little in the different seasons; the deaths from the latter class were most numerous in the autumn. In the winter and spring, the number of deaths was much increased by diseases of the nervous system and diseases of the respiratory organs. But the influence of the seasons on different diseases and classes of diseases will be more apparent, if the deaths occurring in winter and spring be added together, and likewise those in summer and autumn; the former being the seasons in which the average mortality is highest, and the latter those in which it is lowest.

*Average Number of Deaths in each Season in the ten Years* 1845-54.

| Causes of Death. | Winter. | Spring. | Summr. | Autm. | Winter and Spring. | Summer and Autm. | Whole Year. |
|---|---|---|---|---|---|---|---|
| 1. Zymotic diseases .. | 13·7 | 17·3 | 14·6 | 22·7 | 31·0 | 37·3 | 68·3 |
| 2. Dropsy, cancer, &c... | 5·4 | 6·1 | 4·4 | 3.8 | 11·5 | 8·2 | 19·7 |
| 3. Tubercular diseases | 21·6 | 23·8 | 20·5 | 20·8 | 45·4 | 41.3 | 86·7 |
| 4. Disease of brain, &c. | 10·8 | 13·9 | 8.5 | 9·5 | 24·7 | 18·0 | 42·7 |
| 5. Dis. of heart, &c. .. | 4·1 | 4·1 | 1·8 | 2·7 | 8·2 | 4·5 | 12·7 |
| 6. Dis. of lungs, &c. .. | 12·1 | 16·1 | 5·1 | 6·5 | 28·2 | 11·6 | 39·8 |
| 7. Dis. of stomach, &c. | 5·4 | 5·5 | 5·2 | 5·7 | 10·9 | 10·9 | 21·8 |
| 8, 9. Dis. of kidneys & generative organs | 1.6 | ·9 | 1·9 | 2·0 | 2·5 | 3·9 | 6·4 |
| 10. Dis. of joints, &c. 11. Dis. of skin, &c. .. | ·7 | ·5 | ·7 | ·4 | 1·2 | 1·1 | 2·3 |
| 12. Malformations 13. Infantile debility 14. Atrophy .. | 6·6 | 7·9 | 5·7 | 6·2 | 14·5 | 11·9 | 26.4 |
| 15. Senile debility...... | 9·6 | 8·0 | 6·5 | 6·2 | 17·6 | 12·7 | 30·3 |
| 16. Sudden............ | ·3 | — | — | ·3 | ·3 | ·3 | ·6 |
| 17. Violence, &c. ...... | 3·4 | 3.4 | 4·5 | 3·5 | 6·8 | 8·0 | 14·8 |
| 18. Small-pox ........ | ·5 | 1·3 | ·7 | — | 1·8 | ·7 | 2·5 |
| 19. Measles .......... | 1·3 | 1·6 | 1·2 | 3·5 | 2·9 | 4·7 | 7·6 |
| 20. Scarlatina ........ | ·6 | ·2 | ·6 | ·6 | ·8 | 1·2 | 2·0 |
| 21. Hooping-cough .... | 2·8 | 4·8 | 1·8 | 1·5 | 7·6 | 3·3 | 10·9 |
| 22. Diarrhœa.......... | 1·3 | 1·5 | 3·5 | 6·2 | 2·8 | 9·7 | 12·5 |
| 23. Fever ............ | 2·8 | 2·7 | 1·8 | 3·6 | 5·5 | 5·4 | 10·9 |
| 24. Phthisis .......... | 15·9 | 17·5 | 14·5 | 14·0 | 33·4 | 28·5 | 61·9 |
| 25. Hydrocephalus .... | 1·8 | 3·2 | 2·8 | 3.2 | 5·0 | 6·0 | 11·0 |
| 26. Apoplexy .......... | 3·1 | 3·9 | 3·2 | 3·1 | 7·0 | 6·3 | 13·3 |
| 27. Convulsions........ | 4·8 | 5·5 | 2·1 | 2·8 | 10·3 | 4·9 | 15·2 |
| 28. Disease of heart, &c. | 3·7 | 3·9 | 1·7 | 2·5 | 7·6 | 4·2 | 11·8 |
| 29. Bronchitis ........ | 4·4 | 6·3 | 1·3 | 1·5 | 10·7 | 2.8 | 13·5 |
| 30. Pneumonia ........ | 5·2 | 6·9 | 2.8 | 3·8 | 12·1 | 6·6 | 18·7 |
| Causes not specified, &c. | ·1 | ·5 | ·1 | ·1 | ·6 | ·2 | ·8 |

### REPORTS ON THE WATER SUPPLY OF LONDON.

London being now generally supplied with water derived from new sources, we have thought it necessary to undertake an inquiry into the character of the waters furnished by the various companies. These reports will be steadily continued in this Journal, and will form an interesting feature in its pages. In a sanitary point of view nothing is more important than a free and pure water supply; for water is so generally required, that, if it is not a wholesome, it is little less than a poisonous article. The evidence is now also becoming too strong to be resisted, that water may be a medium for the propagation of cholera, yellow fever, and other epidemic diseases.

It is right to say in regard to the London water companies, that they have acted in a prompt and public spirited manner, in carrying out the instructions of the government for obtaining water from new sources. If they have not yet succeeded in freeing London water entirely from all organic impurities, the fault is not strictly theirs. London water is no longer directly contaminated with London sewage, the companies having obtained their supplies from a point above Kingston-on-Thames. Still the sewage, in whole or in part, of Oxford and Reading, two considerable places, flows into the river above Kingston; and though by the current and by evaporation much of the mischief arising from this source is destroyed, it is not easy to suppose that the water reaches Kingston free from all contamination. It is to be hoped and expected that the government will take means to remove this objection to the Thames as a drinking stream.

### I. WEST MIDDLESEX COMPANY.

The West Middlesex Water Company originally obtained their supply from the Thames near to Barnes. They have now, in accordance with the government regulations, taken for their source a point six miles above Teddington Locks, and fifteen miles above the former situation. The present high-level reservoir of the company is at Barrow Hill, and is one hundred and seventy-seven feet above the level of the Thames; but a new reservoir, at a height of about two hundred and eighty feet above the Thames, will shortly be constructed. This will more than meet the government regulation, which fixes on two hundred feet as the minimum. The reservoir at Barrow Hill is covered in, and the supply to the highest levels is, *pro tem.*, secured by engine power.

The subsiding reservoirs and the filtering beds are at Barnes. There are two reservoirs, which, together, cover twelve acres; and three filters, of one acre and a half each. The filter is made of fine Erith sand and gravel. The water is brought from the river above Hampton to the subsiding reservoir at Barnes, through a thirty-six-inch iron main, which extends for eight miles and a half.

*Analysis.*—An analysis gave the following results. The specimen was removed from the reservoir at Barnes, into which it flows after filtration. The colour is clear; the taste sufficiently pleasant; odour, none; degree of hardness by the soap test, 12°. By evaporating an imperial gallon, 18·8 grains of solid residue were obtained. The following tables give the exact composition of the elementary substances contained in the water, and the compounds they form.

| | | | | |
|---|---|---|---|---|
| Silicia | 0·650 | *or* | Silicia | 0·650 |
| Organic matter and water of composition | 2·500 | | Organic matter and water of composition | 2·500 |
| Oxide of iron, alumina, and phosphoric acid | 0·500 | | Oxide of iron, alumina, and phosphoric acid | 0·500 |
| Lime | 6·496 | | Carbonate of lime | 10·500 |
| Soda | 0.750 | | Chloride of sodium | 1·382 |
| Potash | 0·600 | | Chloride of potassium | 0·225 |
| Magnesia | 0·400 | | Sulphate of potash | 0·760 |
| Chlorine | 0·925 | | Sulphate of lime | 1·503 |
| Sulphuric acid | 1.264 | | Carbonate of magnesia | 0·840 |
| Carbonic acid | 9·640 | | | |
| | | | | 18·860 |
| | | | Carbonic acid gas | 4·580 |
| | | | | 23·440 |

Reviewing this analysis, the amount of saline matters present seems moderate; and its presence is an advantage in cases where the water has to pass through leaden pipes, since water holding no salts in solution dissolves lead. The chief salt is the carbonate of lime, which is held in solution by the carbonic acid when the water is cold. On boiling the water the carbonic acid escapes, and the lime salt is precipitated in the form of insoluble carbonate, leaving as saline matter in solution 8·36 grains. Owing to the small proportion of sulphate of lime, the water is remarkably free from hardness, much more so than ordinary well waters, though there is rather less freshness than in spring waters, which are more freely charged with carbonic acid. The sulphate of lime is useful in small proportions, by preventing the solution of lead. The proportion of organic matter given in the above analysis is as small as could well be expected, and if it is never more there is no danger from it.

Taking it all in all, we consider the West Middlesex water a fair specimen of what is required for household purposes. It is pleasant to drink, is moderately soft, deposits a large amount of its saline matter by boiling, and may be transmitted through leaden tubes with safety.

The company supply water over an extensive district, viz., to parts of St. Pancras, Marylebone, Paddington, Kensington, Hammersmith, and Chiswick. Some water is also supplied by the same company to Chelsea, Fulham, Hampstead, and St. Margaret's, Westminster. The total amount of water supplied by this company, for the seven days of the week ending September 30th, 1855, was nearly 43,400,000 gallons, or 6,200,000 gallons per day.

